# Revealing the genetic diversity in the global *ex situ* sweetpotato collection with next generation sequencing technologies

**DOI:** 10.3389/fpls.2026.1864624

**Published:** 2026-09-21

**Authors:** Noelle L. Anglin, Ronald Robles, Genoveva Rossel, Logotonu Meleisea Waqainabete, Semi Cakaunitavuki, Amit Sukal, Norma C. Manrique-Carpintero, Ana Panta, Rainer Vollmer, Alexandre F. S. Mello, Vania C. A. Renno, David Ellis

**Affiliations:** 1 International Potato Center (CIP), Lima, Peru; 2 Centre for Centre for Pacific Crops and Trees (CePaCT), Suva, Fiji; 3 Embrapa Vegetables, Brasília, DF, Brazil

**Keywords:** core collection, DArTseq, *Ipomoea batatas*, phylogenetic tree, population structure

## Abstract

The *in trust* sweetpotato accessions housed at the International Potato Center, Lima, Peru (CIP) represent a large genetic resource collection of this important staple crop, with almost 6, 000 cultivated accessions and 904 crop wild relatives (CWRs). The entire cultivated collection (5,930 accessions of which 22 were tetraploids), was genotyped using high-density Diversity Arrays Technologies sequencing (High Density DArTseq) producing 110,428 SilicoDArT markers (presence-absence variation, PAV) and 94, 096 single nucleotide polymorphic (SNPs) markers. After filtering the data, 57,715 SilicoDArTs and 20,101 SNPs were used for analysis. Genetic identity, diversity, and population structure within the sweetpotato germplasm collection were assessed. Phylogenetic analyses revealed the intraspecific relationships within the entire *ex situ* sweetpotato collection maintained at CIP. High redundancy was observed in materials from Peru and Latin America, matching and confirming morphological assessments and previous data from SSR markers. Based on the SilicoDArT marker data and a cutoff threshold of ≤97.5% genetic similarity, approximately 3,431 accessions (58.07% of the cultivated collection) are genetically distinguishable (non-redundant) accessions. This suggests that much of the collection is highly duplicated or genetically similar and could potentially be archived to save resources, as clonal collections are expensive to maintain. Population structure analyses indicated support for four ancestral populations and revealed low levels of genetic admixture among the inferred populations. This was especially true for germplasm derived from Peru and Africa. A core collection of 342 accessions was constructed from the 3,431 non-redundant subset (representing 9.97% of this subset). Additionally, material from the sweetpotato collection at the Centre for Pacific Crops and Trees (CePaCT) in Fiji was genotyped to rationalize the material between the CePaCT and CIP germplasm collections. Some genetic redundancy was also revealed in the CePaCT collection, with accessions bearing the same name but originating from different sources, having identical fingerprints via DArTseq markers. To the authors’ knowledge, this is the first report of high-density genotyping of an entire cultivated sweetpotato germplasm collection.

## 1 Introduction

Sweetpotato is an important global crop due to its versatility as a food source, with both the roots and leaves being consumed by humans, in addition to its use in industrial products, fodder, and fish food. Various food products are made from the roots, such as starch, flour, noodles, colorants, candy, chips, and alcohol (Lebot, 2008). In 2018, over 91 million tons of sweetpotatoes were produced worldwide across more than eight million hectares of land, with China being the largest producer (FAOSTAT, 2025).

Cultivated sweetpotatoes are classified as *Ipomoea batatas* in the family *Convolvulacea*e. The genus *Ipomoea* is large, and due to its size, sweetpotato and its close relatives are grouped in section *Batatas*, which originally consisted of 13 taxa (Austin, 1978). The sweetpotato genome is characterized as hexaploid (2n = 6x = 90), although there are reports of the existence of tetraploid (2n = 4x = 60) *I. batatas* or feral *I. batatas* (Austin, 1988; Bohac et al., 1993). Due to the polyploid nature of sweetpotato, Mendelian genetics and segregation ratios are complex (Lebot, 2008), with high polymorphic variation. Although several hypotheses about the origin of sweetpotato have been proposed, its exact origin and domestication remain uncertain. A previous report suggested that *I. trifida* and *I. triloba* were the closest relatives of cultivated sweetpotato, with sweetpotato possibly arising from unreduced gametes following hybridization between these wild species (Austin, 1988). Others have reported 2n pollen production of *I. trifida* and assessed the fertility of synthetic hexaploids, leading to the hypothesis that sweetpotato may have originated from hybridization and unreduced gametes between *I. trifida* and a tetraploid *I. batatas* (Orjeda et al., 1990; Freyre et al., 1991). More recently, a study suggested that *I. trifida* was the sole progenitor of sweetpotato, with an autopolyploid origin (Muñoz-Rodríguez et al., 2018). Other studies have proposed an allo-autohexaploid origin (2n = 6x = 90) origin for sweetpotato, with a B_1_B_1_B_2_B_2_B_2_B_2_ genome, produced by crossing a tetraploid and a diploid, followed by whole genome duplication approximately 341, 000 years ago (Yang et al., 2017). The same authors also assumed that the diploid progenitor of cultivated sweetpotato was *I. trifida*, but they reported the tetraploid progenitor as unknown (Yang et al., 2017). Another study of *COSII* genes suggested that sweetpotato is neither a true autopolyploid nor allopolyploid, but instead a segmental allohexaploid, which could explain why bivalent and multivalent chromosome pairing is observed in sweetpotato (Gao et al., 2020).

The main purpose of an *ex situ* genebank is to preserve and safeguard genetic diversity of important crops and their wild relatives. Clonal genebanks comprise unique accessions compared to seed genebanks because accessions are static, with a fixed combination of alleles preserved over time, whereas seed accessions are stored as populations with alleles that are in flux (genetic drift). Thus, in typical allogamous seed collections, each accession potentially contains a new combination of alleles that confer specific traits and adaptive potential (McCouch et al., 2012). Access to genetic diversity held in genebanks is a key component for breeders to improve cultivated crops and ensure food security. Frequently, elite lines continue to be improved, while landraces often remain untapped, uncharacterized, and underutilized (Lee, 1998). Generally, genebanks preserve as much unique diversity as possible (via collection or donation) for their crops of interest. This means that total collection sizes can be quite large and often not characterized thoroughly for traits of interest, limiting a user’s ability to strategically select genotypes/phenotypes for specific purposes. As a result, only a small proportion of the diversity held by genebanks tends to be utilized or intensely differentiated, because genebank resources are focused on conservation and keeping the material alive, rather than on trait evaluation especially traits important for breeding such as yield, disease resistance, and quality traits. Therefore, many collections have not been well evaluated for important agronomic traits, especially in collections that include several thousand accessions. Categorizing *I. batatas* based on morphological descriptors into varietal groups has proven to be quite difficult due to continuous variation (Roullier et al., 2011) and residual genotype by environment interaction (GxE) effect.

The genebank at the International Potato Center (CIP) in Lima, Peru, houses one of the largest sweetpotato collections, containing almost 6, 000 accessions of cultivated sweetpotato. These accessions are held *in trust* under the International Treaty on Plant Genetic Resources for Food and Agriculture (ITPGRFA) and are distributed to requestors worldwide for research, breeding, and education for food and agriculture. Originally, the germplasm was maintained in the field, but this presented multiple challenges, including exposure to pests and diseases, variable environmental conditions, attrition of accessions due to planting a limited number of roots, mixing of accessions, mislabeling, or mishandling of accessions, contamination of plots by volunteer plants due to lack of rotation, and high maintenance costs. These factors contribute to the undermining of both maintenance and genetic identity within a genebank (Huaman, 1999; Jarret et al., 2019). As a result, the cultivated material is now maintained as *in vitro* clones for secure medium-term conservation. The germplasm in the collection is derived from 66 countries and consists of landraces, breeding lines, and improved varieties. A total of 27.8% of the CIP collection (1, 621 accessions) originated or was developed in Peru. The overarching goal of this project was to generate genetic fingerprints for the entire *ex situ* collection of sweetpotato at CIP using next-generation sequencing technologies. As part of the project, CIP collaborated with the Centre for Pacific Crops and Trees (CePaCT) in Fiji to include a subset of Pacific sweetpotato accessions for comparative analysis between collections. CePaCT, hosted by the Pacific Community (SPC), serves as the regional genebank for Pacific Island Countries and territories and conserves sweetpotato and other crop collections *in trust* on behalf of SPC member countries, as a regional public good, to support conservation, research, training, and sustainable use. The objectives of this research were (i) to evaluate the genetic diversity of the entire cultivated sweetpotato collection using high-density (HD) reduced representation sequencing/genotyping via DArTseq markers, (ii) to evaluate phylogenetic relationships and population structure of the collection, (iii) to identify putative duplicates in the collection, (iv) to compare the CIP collection with a subset of sweetpotato accessions (188) from the CePaCT genebank in Fiji, and (v) to construct a core collection representing the majority of genetic diversity with minimal redundancy for sweetpotato researchers.

## 2 Materials and methods

### 2.1 Plant material, DNA extractions and high density DArTseq genotyping

Young leaves from greenhouse-maintained plants or *in vitro* shoot cultures were collected from the CIP sweetpotato genebank. The cultivated collection is clonally maintained; therefore, each cultivated accession is represented by a single genotype, from which one DNA sample was obtained for DArTseq genotyping. In contrast, tetraploid wild *Ipomoea* accessions are conserved as seed populations. Although each seed population constitutes a single accession, it contains multiple individual genotypes. Consequently, depending on availability, tetraploid accessions were represented by one or more DNA samples derived from different individuals. In total, the study included 5, 970 DNA samples, comprising 5, 908 DNA samples representing cultivated hexaploid accessions and 62 DNA samples representing 22 tetraploid accessions (**Supplementary Table 1**).

DNA was extracted using the CTAB method with modifications (Doyle and Doyle, 1990). Approximately 100 mg of leaf tissue was collected in an Eppendorf^®^ microcentrifuge tube containing 700 μL of 2X CTAB extraction buffer (CTAB 2%, EDTA 0.5 M pH 8, NaCl 5 M, Tris-HCl 1 M pH 8, PVP 1% and Mili Q water) along with 2 μL of β-mercaptoethanol. Ceramic beads were added to the microcentrifuge tubes, and the leaves were ground using a FastPrep^®^-24 (MP Biomedicals, SA, CA, USA) for one minute. The resulting homogenate was incubated for 45 min at 65 °C, and then 750 μL chloroform:isoamyl alcohol (24:1) was added. The tubes were gently inverted for five min and then centrifuged at 14, 000 rpm for five min. The aqueous phase was transferred to a new tube containing 70 μL of 10X CTAB. Another 750 μL of chloroform:isoamyl alcohol [24:1] was added to this mixture, and centrifugation was repeated to separate the phases. The upper phase was transferred to a new tube, to which an equal volume of cold isopropanol was added, and the mixture was incubated at -20 °C for 15 min. The tube was then centrifuged at 14, 000 rpm for five minutes, and the pellet was washed with ethanol. The pellet was dried and resuspended in 100 μL of TE buffer (pH 8), and 1 μL RNase (10 mg/mL) was added. The DNA was incubated at 37 °C for 2 hours to allow resuspension of the pellet. The quality and quantity of the DNA were determined by running 1 μL on a 1% agarose gel with lambda DNA digested with PstI as a standard, and by measuring the concentration on a NanoDrop 1000 Spectrophotometer (Thermo Scientific, Nanodrop Products, DE, USA). DNA concentration was adjusted to ~50–100 ng/μL for DArTseq genotyping.

Samples of all DNA extracts were shipped to Diversity Arrays Technology Pty Ltd, Canberra, Australia, for processing using a reduced representation sequencing method known as DArTSeq™. Approximately 5% of the DNA samples submitted for DArTseq analysis were independently re-extracted to generate biological replicates for estimating the sensitivity and specificity of the Jaccard genetic distance threshold used to classify accession as genetically distinguishable or potentially redundant. Technical replicates were also generated by DArT PL to calculate the reproducibility of each marker. The DArT complexity reduction and next-generation sequencing protocols for data production were previously described (Kilian et al., 2012). Sequencing data were analyzed by DArT PL using DArTsoft14, an automated genotypic data analysis program, and DarTdb, a proprietary management system.

DNA from the CePaCT sweetpotato germplasm collection was extracted according to (Li et al., 2008) with modifications. Approximately 100 mg of leaf tissue was collected and placed in a 2 mL microcentrifuge tube containing a 7 mm steel bead. Two hundred microliters of CTAB extraction buffer (CTAB 2%, EDTA 20 mM, NaCl 1.4 M, Tris-HCl 100 mM pH 8, PVP-40 2%, 2-mercaptoethanol 0.2% in nuclease-free sterile water) was added to the leaf tissue, and the tubes were cooled at -20 °C for 5–15 minutes until the contents were partly frozen. The tubes were then removed from the cooler and homogenized in the TissueLyser (Qiagen, Australia) for 1 min at 30 HZ. The sample holder was inverted, and the homogenization step was repeated. An additional 800 µL of CTAB was added to the tube and mixed well. The homogenate was incubated at 65 °C for 15 min followed by centrifugation at 10, 000 g for 5 min. Approximately 650 µL of the supernatant was transferred to a 1.5 mL microcentrifuge tube. An equal volume of chloroform/isoamyl alcohol (24:1) was added and mixed well. The tubes were centrifuged at 14, 000 rpm for 10 min. Five hundred microliters of the supernatant were transferred to a new 1.5 mL microcentrifuge tube containing 350 µL of isopropanol and mixed well. The subsequent solution was centrifuged at 14, 000 rpm for 10 min. The tubes were decanted, ensuring that the DNA pellet was not disturbed. DNA pellets were washed with 70% ethanol. The ethanol was discarded, and the pellets were dried and resuspended in 100 μL of TE buffer (pH 8). One microliter of RNase (10 mg/mL) was added, and the DNA was incubated at 37 °C for 2 hours. DNA was then shipped directly to Diversity Arrays in Canberra, Australia, for library preparation and reduced representation sequencing.

### 2.2 Data analysis

Genotyping by DArTseq generated two marker datasets: 110,428 SilicoDArT markers and 94,096 SNP markers, all with reproducibility values greater than 95%. SilicoDArT markers were provided as a binary matrix (0/1), indicating whether a specific DArTseq genomic fragment was successfully generated and sequenced in each genotype. As dominant presence/absence markers, they do not distinguish between homozygous and heterozygous states. SNP markers represent single nucleotide polymorphisms identified within these sequenced DArTseq fragments and can therefore only be scored when the corresponding fragment is present. DArTseq reports SNP genotypes using diploid genotype coding; consequently, explicit allele dosage across the six homologous chromosomes of cultivated hexaploid sweetpotato cannot be resolved. Prior to downstream analyses, the marker datasets were subjected to quality-control filtering. For the SilicoDArT dataset, markers were filtered by excluding those with a Call Rate below 95% (the percentage of samples in which a given target could be successfully scored) and those with a marker frequency below 5% across samples. Because of the large difference in the number of DNA samples between the cultivated dataset (5, 908 DNA samples, one sample per accession) and the tetraploid dataset (62 DNA samples representing 22 tetraploid accessions), marker filtering was performed separately for the two groups to prevent informative markers for the tetraploid material from being unnecessarily discarded due to the large imbalance in sample numbers if filtering were applied directly to the complete dataset of 5,970 DNA samples. SNP markers generated through reduced-representation approaches that rely on restriction enzymes typically show higher levels of missing data, as sequencing depends on the presence of restriction sites flanking the SNP position. Accordingly, for the SNP dataset, markers were filtered by excluding those with a Call Rate below 80% and those with a minor allele frequency (MAF) below 2%. These filter parameters pipelines retained a total of 57,715 SilicoDArT markers and 20,101 SNP markers.

A genetic dissimilarity matrix among accessions was calculated using the Jaccard dissimilarity coefficient in DARwin (Dissimilarity Analysis and Representation for Windows) v 6.0.14 (Perrier and Jacquemoud-Collet, 2006) for SilicoDArT and Manhattan distance, using the dartR package (Mijangos et al., 2022) in R v 4.5.0, for SNP data to assess genetic redundancy within the collection, a similarity threshold was used as an operational criterion rather than as direct evidence of clonal identity. Accessions showing genetic similarity above the selected threshold with one or more other accessions were considered potentially redundant for genebank management. Conversely, accessions not exceeding the selected threshold were retained as genetically distinguishable.

Trees were constructed in the DARwin software using the unweighted Neighbor Joining method for both SilicoDArT and SNP data. Phylogenetic trees were annotated using iTOL (Letunic and Bork, 2016) to color-code branches by country of origin. Principal Coordinate Analysis was performed using DARwin (version 6.0.14) software based on the distance matrix. Estimates of redundancy within the germplasm collection were calculated using the distance matrix and the hclust and cutree functions of the stats package in R v 4.5.0.

Diversity metrics were also calculated to evaluate variation among material from different geographic regions. Gene diversity, or expected heterozygosity, is a measure of genetic variation that calculates the proportion of heterozygous genotypes expected under Hardy-Weinberg equilibrium. In other words, gene diversity is the calculated probability that randomly sampled alleles from a population are different (Harris and DeGiorgio, 2017). It was calculated using the following Nei’s formula, where *i* is the number of alleles at a locus and p*_i_* is the frequency of the allele in a population (Nei, 1973):


$$He=1-\;\sum_{i=1}^{I}p_{i}^{2}$$

Similarly, the Shannon index of diversity was calculated according to the formula:


$$H^{\prime }=\;-\;\sum_{j=1}^{S}p_{i}\ln p_{i}$$

The values of the fixation index (Fst) were calculated using the package *Statistical analysis of Mixed Ploidy Populations (StAMPP)* v1.6.1 in R (Pembleton et al., 2013) using the 20,101 SNPs. Polymorphic information content (PIC) was calculated for dominant markers by using the following formula (Roldán-Ruiz et al., 2000):


$$PIC_{i}=2f_{i}\left(1-\;f_{i}\right)$$

where *f_i_* is the frequency of the amplified allele band present, and (1 − *f_i_*) is the frequency of the null allele (band absent) of marker *i*.

Population structure was inferred using STRUCTURE v2.3.4 (Pritchard et al., 2000) from the filtered binary SilicoDArT marker matrix. To minimize the overrepresentation of highly similar genotypes, the analysis was performed using the non-redundant cultivated dataset obtained after applying the selected genetic similarity threshold. Marker filtering was then repeated for this reduced dataset using the same filtering criteria described above, and the resulting filtered marker matrix was used as input for STRUCTURE. This program infers population structure and assigns individuals to populations based on their genotypes. STRUCTURE uses model-based clustering with a Bayesian approach to identify clusters based on a fit to Hardy-Weinberg equilibrium and linkage equilibrium. Multiple runs of STRUCTURE were performed by setting K (the number of populations) from 1-10, producing 10 replications for each K. The burn-in time was set to 10, 000 cycles, and additional Markov Chain Monte Carlo (MCMC) replications were set to 50,000. The ancestry model was set to admixture without using *a priori* population information, allele frequencies were assumed to be correlated, and dominant SilicoDArT markers were analysed with the ploidy parameter set to 1 (haploid mode). Additionally, parameters (alpha, F) across single runs of STRUCTURE were plotted at the different K values to evaluate where the data converged. At low K values (K = 2 – K = 4), convergence was observed after 1000 burn-in iterations. Because finding an optimal K value for datasets can be a challenge, the Evanno method (Evanno et al., 2005) was employed in Structure Harvester to choose the optimal K value (Earl and vonHoldt, 2012). Further, statistics such as consistency in alpha during the run, a proportion of individuals not being symmetric (~1/K), and a low Pr(X/K) value, generated from STRUCTURE runs were also used to confirm the appropriate K value.

### 2.3 Core collection

A core collection was constructed from the 5,930 accessions maintained at CIP Genebank. The first step was to reduce the dataset by removing genetic duplicates and highly similar individuals based on genetic distance, as there was significant genetic redundancy in the germplasm collection. As a result, a total of 3,431 accessions were retained for the construction of the core collection after eliminating the highly redundant material. Core Hunter version 3.2.1 (De Beukelaer et al., 2018) was used to construct the core collection. Genetic distances among accessions were calculated using the Modified Rogers’ distance and the sampling strategy employed was the average entry-to-nearest-entry (AEN) criterion. This strategy aims to maximize the average distance between selected individuals for the core to optimize the selection of most unique and diverse accessions. Additionally, the data were parsed by country of origin and biological status for the selection of the core collection.

## 3 Results and discussion

### 3.1 Collection wide genotyping

The entire cultivated sweetpotato germplasm collection at the International Potato Center (CIP) in Lima, Peru, was genotyped using high-density DArTseq, producing a total of 57,715 SilicoDArTs and 20,101 SNP markers after quality filtering the raw data. The dataset was used to assess the genetic diversity of 5,930 accessions, comprising 5,908 hexaploid cultivated accessions and 22 tetraploid accessions. A phylogenetic tree based on 57,715 SilicoDArT markers was constructed to assess genetic relationships among cultivated accessions. The tetraploids were utilized to root the tree, as they are considered a bridge between wild and cultivated material, frequently referred to as feral *I. batatas* (Austin, 1988; Bohac et al., 1993). All tetraploids grouped together and clustered by geographic origin, with tetraploids from Ecuador separating from Colombian tetraploids. Generally, phylogeography patterns were apparent, with many accessions derived from a specific country or region clustering together (**Figure 1**). However, due to the movement of germplasm, which is sometimes donated by one country but originates in another, clustering by geographic origin was not perfect, and some exceptions were observed. Despite this, most accessions clustered by country of origin, and as genetically redundant material was removed, this trend became clearer and more distinct. Similar clustering patterns were observed when genotyping the entire collection with 20 SSR markers. However, clustering by country of geographic origin was more pronounced with the 57, 715 SilicoDArT markers compared to the SSR markers (Anglin et al., 2021).

**Figure 1:**
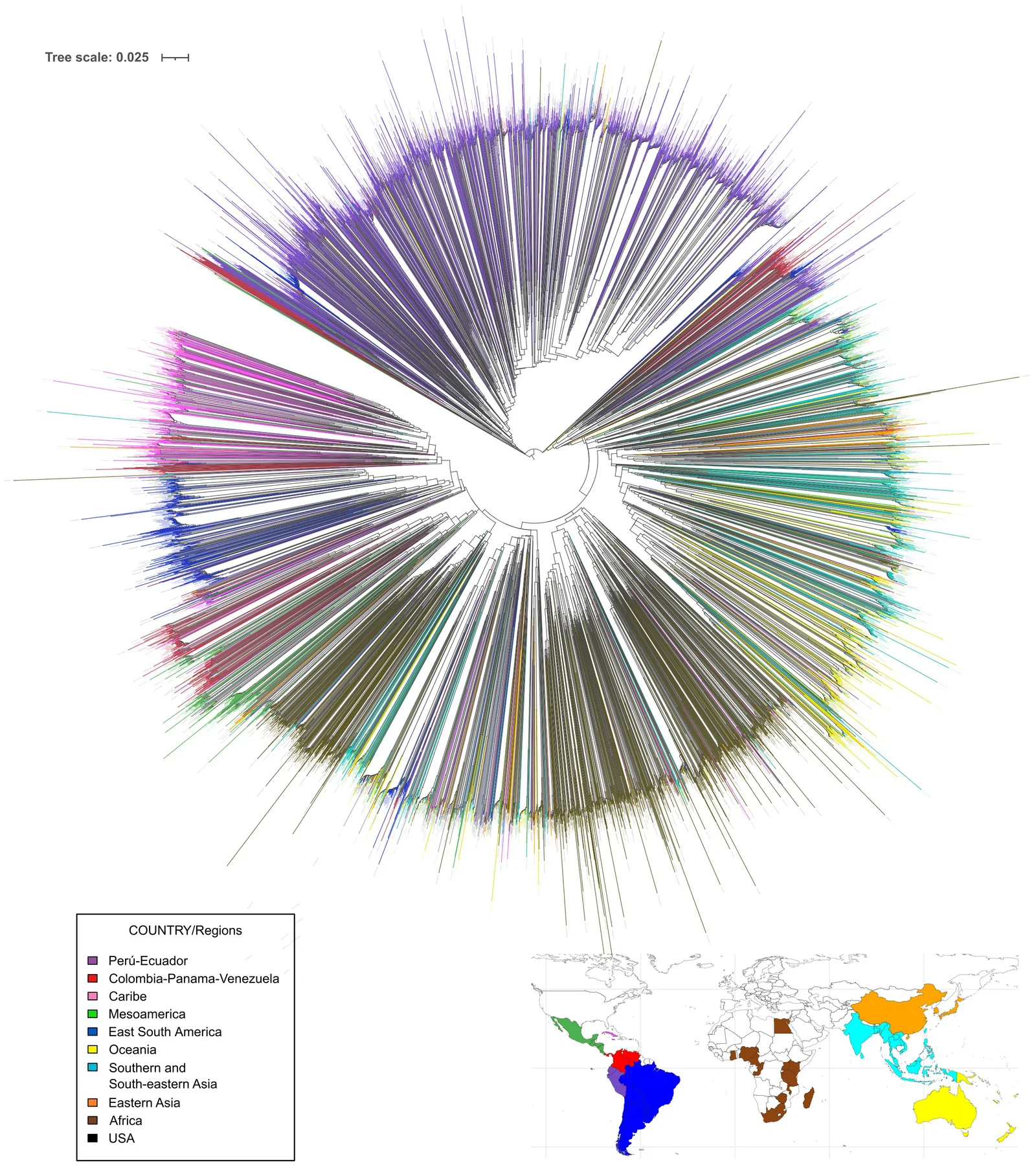
Phylogenetic tree based on 57, 715 SilicoDArT markers of the 5, 930 sweetpotato accessions from the CIP Genebank collection.

Due to the consistent clustering of accessions by geographic origin, diversity metrics were calculated based on the geographic origin of each accession to evaluate diversity within sweetpotato accessions from different regions. Gene diversity, also known as expected heterozygosity, is a measure of genetic variation within a population. It was calculated and shown in boxplots (**Figure 2A**). The average gene diversity did not vary greatly among the various geographic locations, ranging from 0.251 in the Peruvian/Ecuador group to 0.312 in the Asian group, respectively. This low variation in gene diversity could be attributed to challenges in determining allele dosages from the data. Polymorphism Information Content (PIC) was calculated by region and ranged from 0.250 to 0.311, with the Peruvian material showing the lowest PIC value. The Shannon Diversity Index was also calculated using the 57,715 SilicoDArT markers, with the Peruvian/Ecuador material showing the lowest gene diversity of any region analyzed (**Figure 2B**). This lower Shannon Index is likely due to the high genetic redundancy observed in the Peruvian accessions, which has been noted in evaluations using 20 SSR markers (Anglin et al., 2021), morphological characterizations, and here with the high-density DArTseq markers. The remaining populations showed equivalent Shannon index values. Fst values, which indicate the proportion of genetic variation in a subpopulation relative to the total genetic variance, were also calculated (**Table 1**; **Figure 2C**). When Fst values were compared between different geographical origins (**Table 1**), the Peruvian material exhibited the highest differentiation compared to other regions, with the highest Fst being 0.174 between Perú-Ecuador and Mesoamerica groups. Overall, the Fst values in the other geographic regions were not highly variable and were comparable (**Figure 2C**). This suggests that the accessions from Peru are unique compared to those collected or donated from other countries worldwide. This is likely due to the distinct SNP variants found in the Peruvian and Ecuadorian populations relative to other populations. Based on Fst comparisons, material from Africa showed similar genetic variance to that from the United States, Asia, and Oceania.

**Figure 2:**
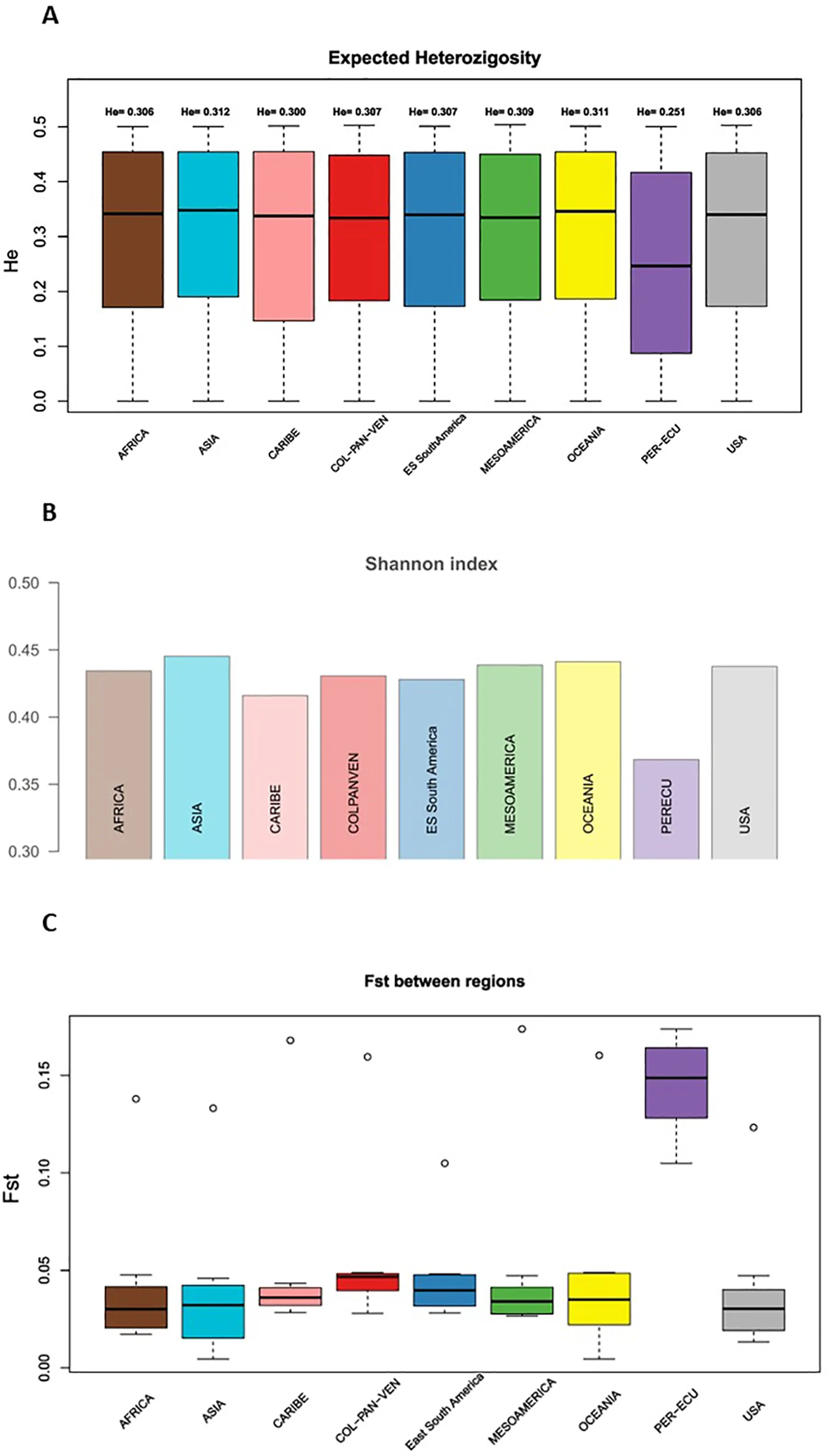
Genetic diversity analysis of sweetpotato CIP Genebank collection using different metrics. **(A)** Box plots of expected heterozygosity/gene diversity by geographical region which was calculated with the 20,101 SNP markers. Average expected heterozygosity for each region is shown above each box plot. **(B)** Shannon diversity index calculated on accessions grouped by geographical origin. **(C)** Fst values calculated by geographic origin.

**Table 1:** Pairwise FST estimates among cultivated sweetpotato geographic groups calculated using the 20, 101 filtered SNP dataset.

	*Africa* *n =1099*	*ASIA* *n =928*	*CARIBE* *n =405*	*COL-PAN-VEN* *n =300*	*East South America* *n =487*	*MESOAMERICA* *n =215*	*OCEANIA* *n =488*	*PER-ECU* *n =1782*	*USA* *n =187*
Africa									
ASIA	0.0172								
CARIBE	0.0285	0.0386							
COL-PAN-VEN	0.0477	0.0459	0.0367						
East South America	0.0318	0.0369	0.0317	0.0426					
MESOAMERICA	0.0353	0.0274	0.0354	0.0280	0.0473				
OCEANIA	0.0234	0.0046	0.0434	0.0489	0.0481	0.0266			
PER-ECU	0.1379	0.1331	0.1680	0.1595	0.1049	0.1738	0.1602		
USA	0.0176	0.0133	0.0324	0.0473	0.0282	0.0328	0.0209	0.1232	

COL-PAN-VEN, Colombia, Panama, and Venezuela; PER-ECU, Peru and Ecuador.

n, Number of cultivated accessions included in each geographic group.

*17 accessions without unknown country of origin or with low representativeness were not included in this analysis.

FST values are presented as descriptive estimates and were not subjected to statistical significance testing.

Evidence of genetic redundancy in the sweetpotato germplasm collection has been observed in previous genotyping work with 20 SSR markers (Anglin et al., 2021), routine morphological assessments, and the DArTseq data, which provides a broader genome coverage than SSR markers. **Figure 3** illustrates two accessions that were genetically similar by SSR markers, DArTseq, and morphological traits, and clustering together with no genetic variation in the phylogeny. The DArTseq data was thus used to estimate the proportion of unique material in the collection. The pairwise distance matrices using SilicoDArTs and SNP markers were plotted to assess divergence within the collection (**Figure 4**). Using SilicoDArT markers, pairwise genetic distances were analyzed with cutoffs at 95% and 97.5% of similarity, revealing relatively few genetically unique individuals. At a 95% similarity threshold, 2, 903 accessions were retained as genetically distinguishable, whereas at a 97.5% similarity threshold, 3,431 accessions were retained. These represent 49.1% and 58.1% of the cultivated collection, respectively, as non-redundant accessions under the selected similarity thresholds, confirming previous findings (via SSRs and morphological assessments). Thus, a significant portion of the collection is genetically and morphologically similar. Many of these accessions are likely siblings, derivatives from breeding programs, material obtained from sources with different names but identical clones, or groups of accessions from geographically close regions, showing little genetic variation. This is reflected in the Fst values, the short branches in the phylogenetic tree, and the similar genetic distances among pairs of individuals (**Figures 1**, **2**, **4**).

**Figure 3:**
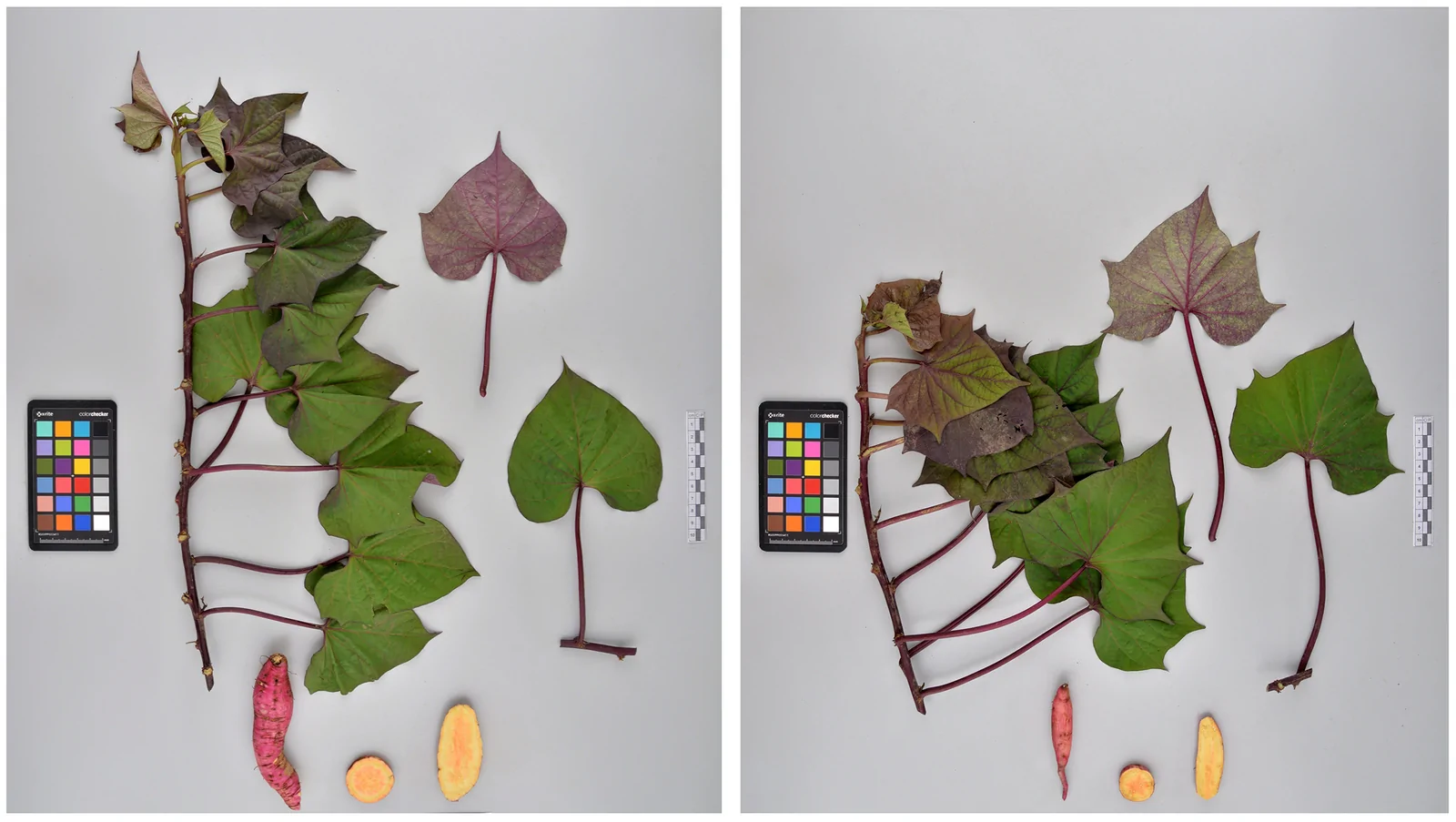
Example of two sweetpotato accessions from the CIP genebank collection: CIP 401112 (left) and CIP 401114 (right), both derived from Guatemala, which had identical genotypes by SSR, DArTseq, as well as identical phenotypes in morphological characterization.

**Figure 4:**
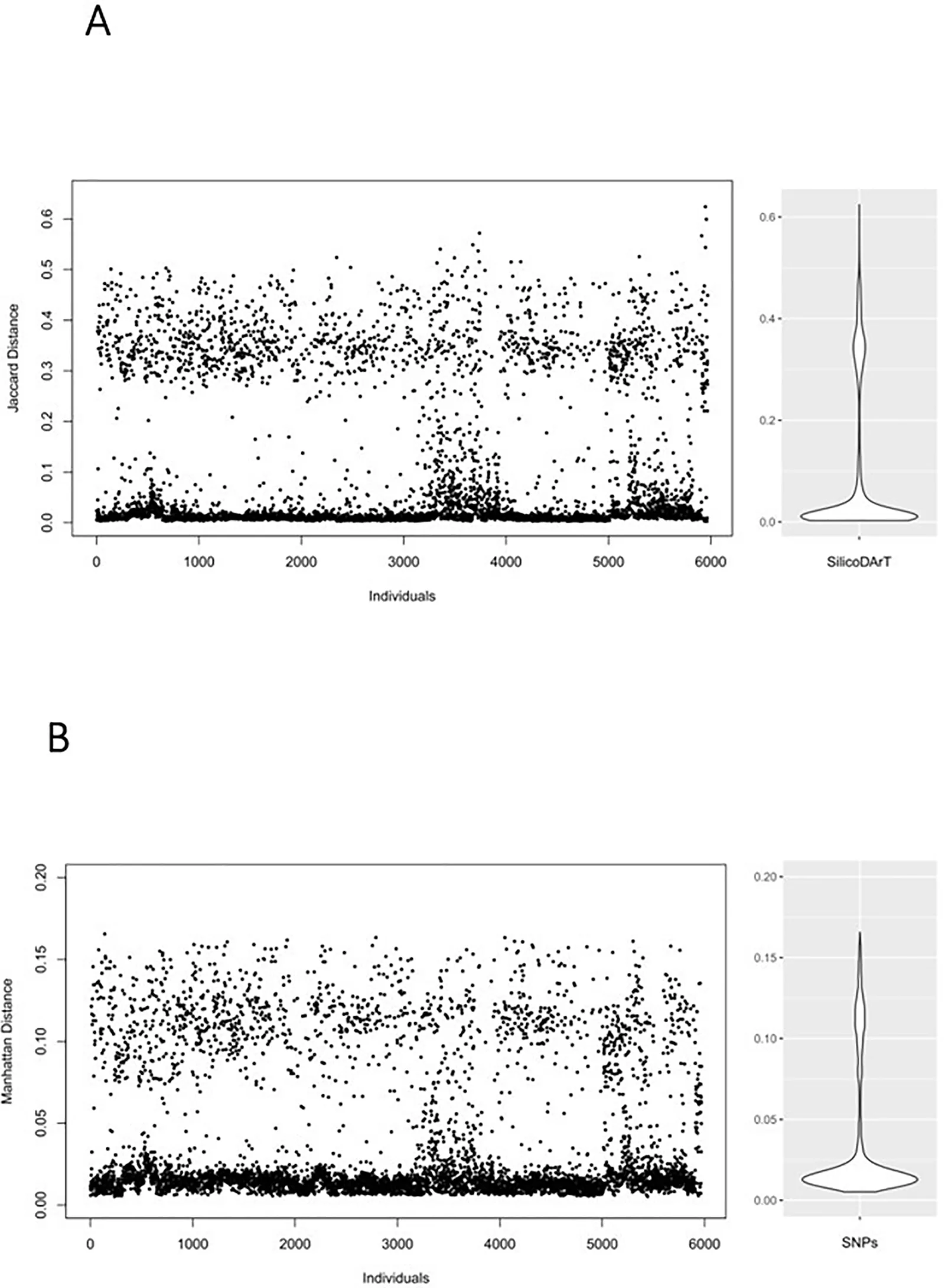
Pairwise genetic distances of all 5,930 sweetpotato accessions at CIP, using **(A)** SilicoDArTs (presence/absence) and **(B)** SNP markers produced from the DArTseq method. The Y-axis represents the dissimilarity distances, and the X-axis shows individual pairs of accessions. Dots near zero indicate little to no genetic divergence. A violin plot was constructed using the same data to illustrate the significant proportion of the collection with high similarity among individuals (increased density in the violin below 0.025, which corresponds to 97.5% of similarity), based on the genetic distance of the DArTseq markers.

The dataset for the cultivated sweetpotato accessions was reduced to include only accessions with a genetic similarity of ≤97.5%, removing the most genetically redundant accessions. A phylogeny was constructed for the 3,431 unique cultivated accessions using 27,917 SilicoDArT markers and 18,636 SNP markers (**Figures 5**, **6**). These marker sets resulted from reapplying the same marker filtering criteria used previously, but this time restricted to this subset of cultivated accessions. Material from Africa split into two main clusters: one representing breeding lines donated by the International Institute of Tropical Agriculture (IITA) and a second, smaller cluster of African landrace accessions. The genetic pools of African landraces and improved African breeding lines were markedly distinct, appearing widely separated on the phylogenetic tree. As in previous analyses, the phylogeny showed that material from Peru and Ecuador was clearly differentiated from the other accessions. Minor differences were noted between the phylogenies produced using the two marker types (**Figures 5**, **6**). The SNP markers clustered accessions from East South America with Asia and Oceania groups, African landraces clustered with Asia, and Caribbean accessions from Jamaica with those from Mesoamerica. Overall, accessions tended to be grouped by country of origin, and clustering patterns similar to those observed for the entire collection were observed.

**Figure 5:**
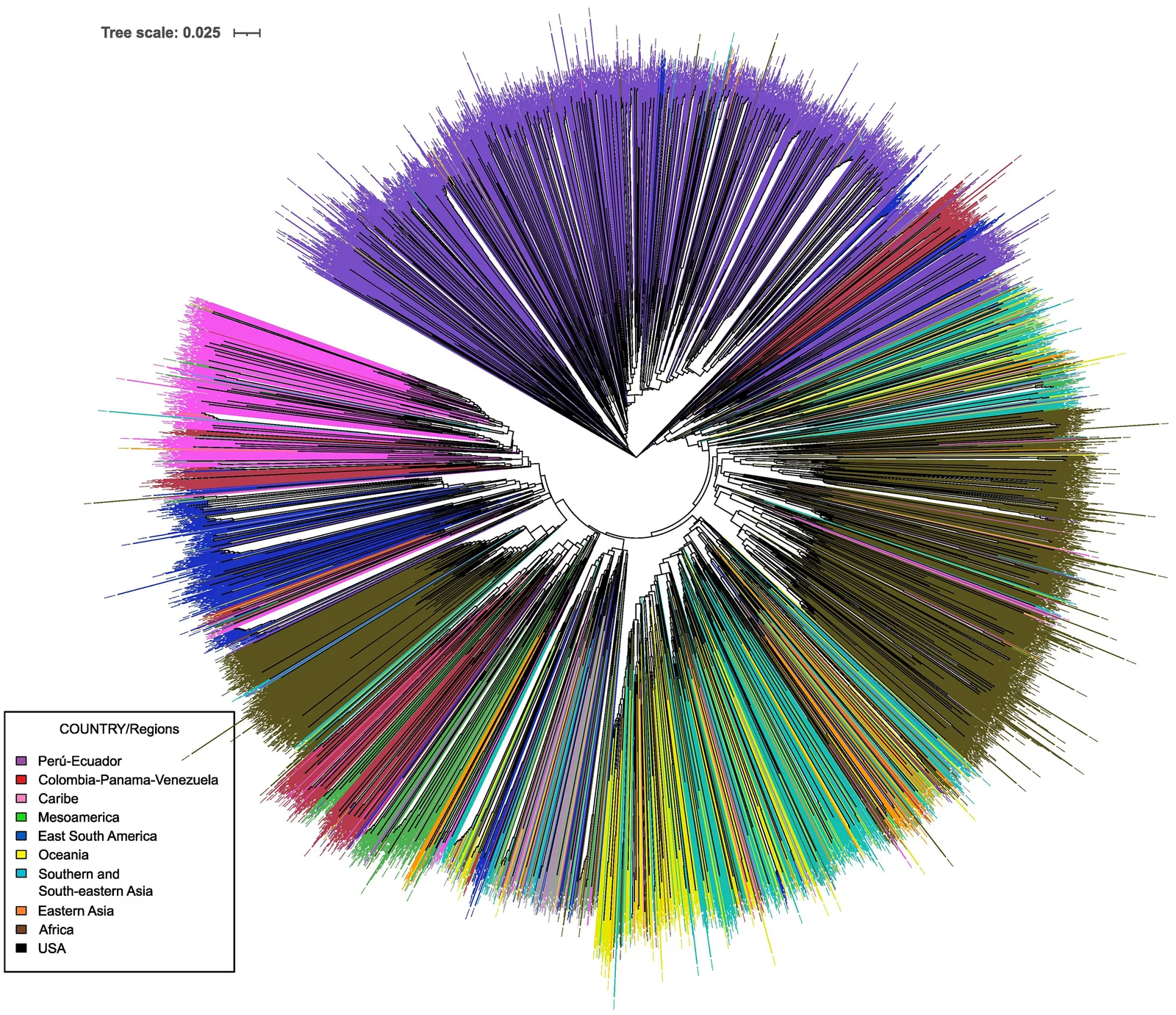
Neighbor-joining tree of 3,431 non-redundant accessions at CIP, color-coded by country of origin using 27, 917 SilicoDArT markers.

**Figure 6:**
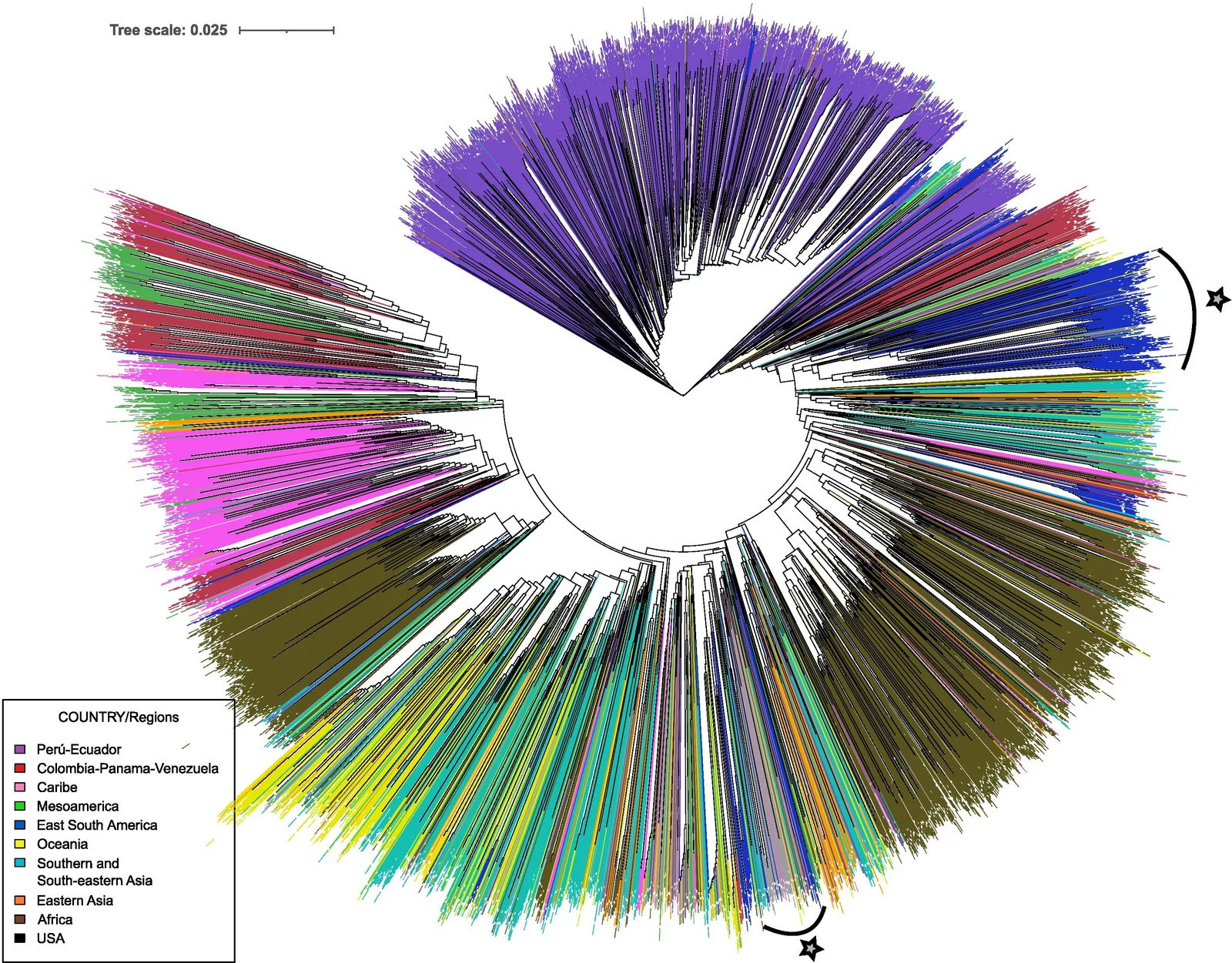
Circular phylogeny of 3,431 accessions obtained using 18,636 SNP markers. Stars represent minor changes in clustering between the phylogeny based on SilicoDArT markers and the one based on SNP markers.

Principal Coordinate Analysis (PCoA) was also calculated for the 3,431 unique cultivated accessions together with tetraploids using SNP markers (**Figure 7**), in which axis 1 explained 20.03% of the total variation and axis 2 accounted for 3.34% of the total variation. This analysis clearly demonstrates the Peruvian material is differentiated from the other populations. The tetraploids, marked as squares in **Figure 7**, appear to be distinct from the other accessions of the same geographical origin. However, the differences in ploidy levels, with most accessions being hexaploids and only a few being tetraploids in this dataset, may introduce biases that make these individuals appear different from other accessions within the same geographic origin. Additionally, PCoA revealed a clear divergence between landraces and breeding lines from Africa, although the divergence between Oceania and Asia accessions was not evident in either the PCoA or the phylogenetic analyses.

**Figure 7:**
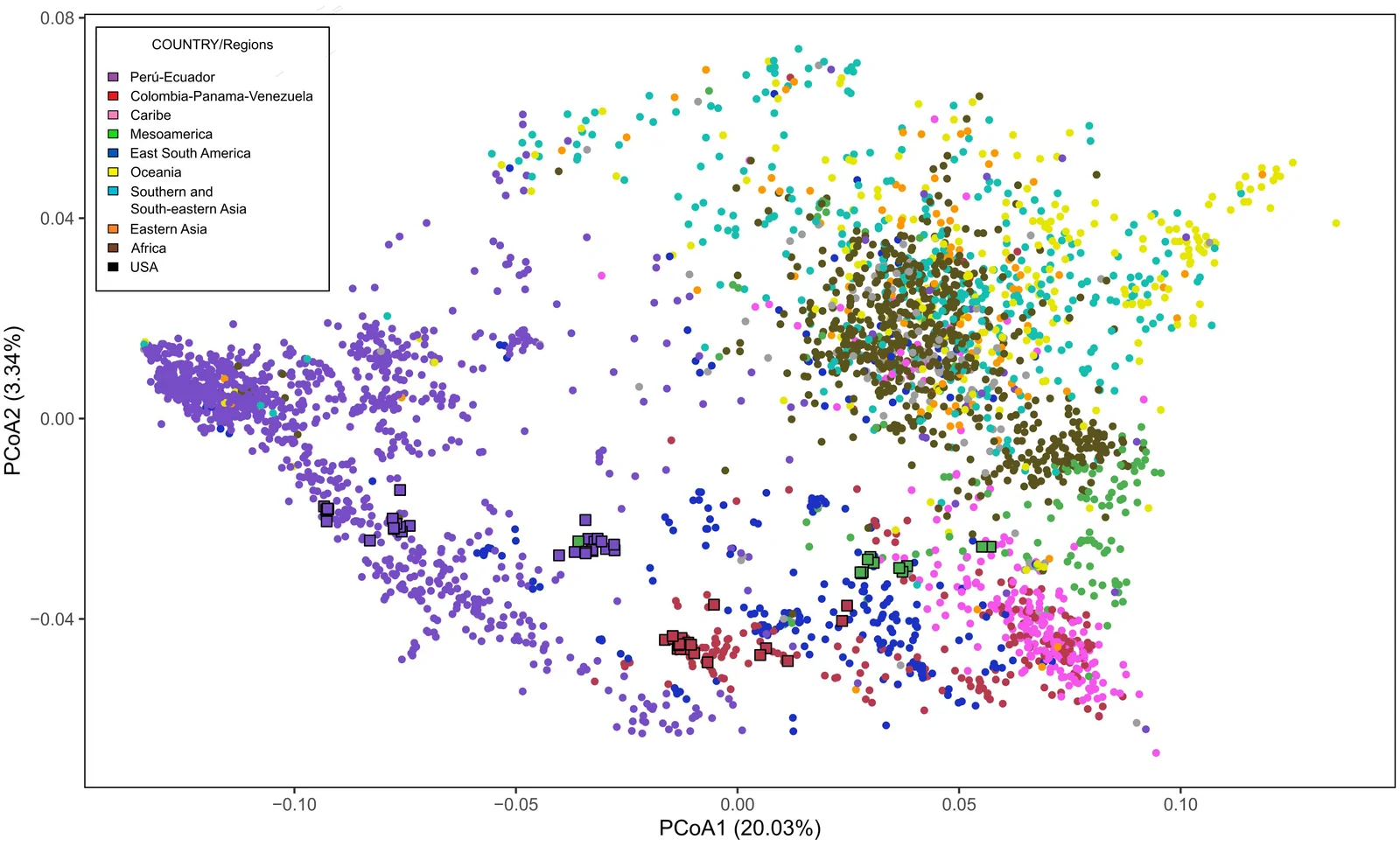
Principal Coordinate Analysis (PCoA) based on SNP markers of the non-redundant set of 3,431 cultivated accessions together with tetraploids. Tetraploid samples are represented by squares, while the remaining hexaploid accessions are represented by circles.

Population structure was estimated using STRUCTURE for the non-redundant cultivated dataset, comprising 3,431 accessions. After reapplying the marker-filtering criteria to this subset, the resulting dataset contained 27,917 filtered SilicoDArT markers, which were used for the STRUCTURE analysis (**Figure 8**). Strong support was observed for four populations: one comprising the Perú- Ecuador accessions, a second comprising accessions from the Caribbean, Mesoamerica, and Eastern South America, a third with African landraces along with material from Oceania and Asia, and a fourth representing African breeding lines and material from Asia. More admixture was observed among the sweetpotato accessions with DArTseq data than was seen in previous genotyping studies using 20 SSR markers (Anglin et al., 2021), likely due to the increased marker density, which provided more genetic information. Both SSR markers and DArTseq markers used on this collection suggested four populations in STRUCTURE, confirming previous findings (Anglin et al., 2021). The Peruvian material tended to have the least admixture at any K value, with very little shared ancestry from other populations, suggesting it has been more genetically isolated compared to the other groupings. The two populations containing material from Africa, Oceania, and Asia exhibited the most admixture at all K values, indicating more genetic exchange within this material.

**Figure 8:**
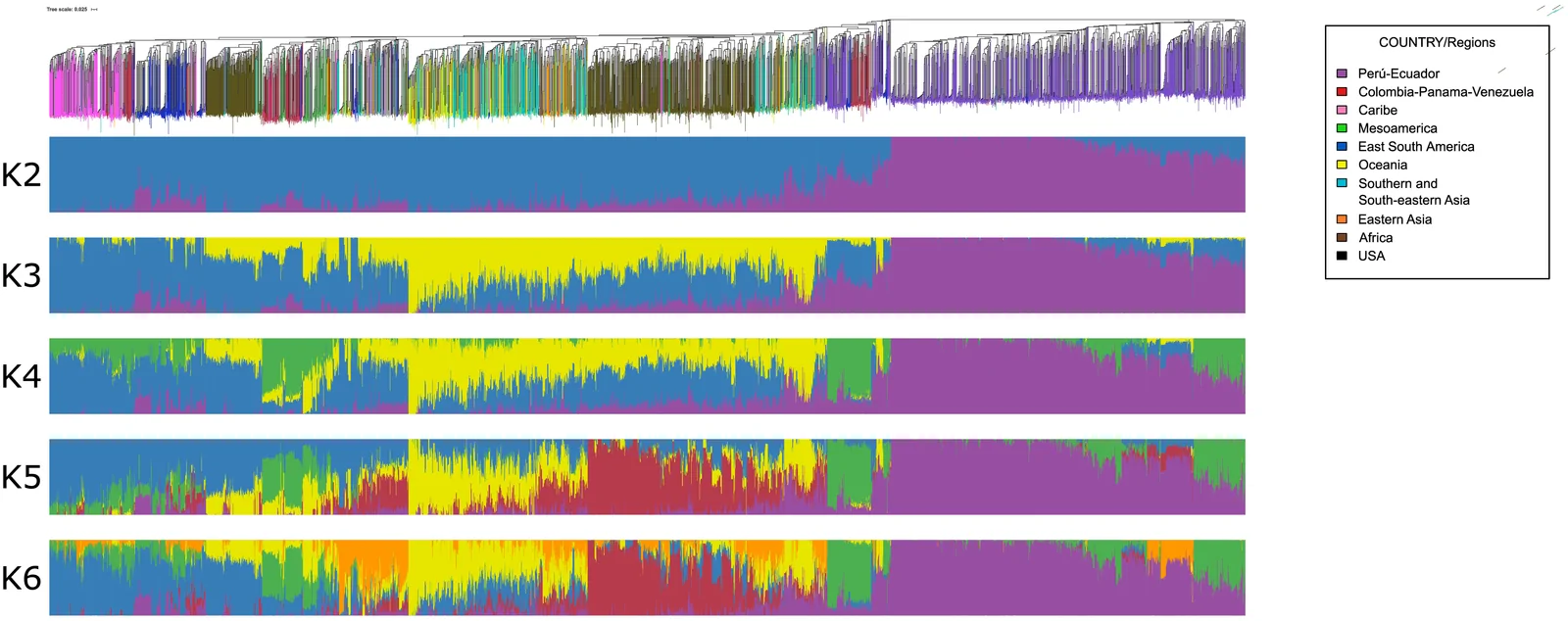
Population structure data K = 2–6 of SilicoDArTmarkers for the non-redundant set of 3,431 accessions in the *ex situ* collection at CIP. First panel is K = 2 and last panel is K = 6.

### 3.2 Comparison between collections

A subset of 188 sweetpotato accessions from the genebank at CePaCT in Suva, Fiji, primarily originating from the Pacific and Asia regions, was also genotyped using low density DArTseq. A phylogenetic tree of the CePaCT accessions alone is presented in **Figure 9B**. To assess the genetic relationships between both collections, these accessions were subsequently analyzed together with all 5,930 genotypes from the CIP collection (**Figure 9A**), allowing the identification of genetically distinguishable and potentially redundant germplasm shared between the two collections. Several accessions from the CePaCT collection were highly similar and likely represent genetic duplicates or closely related lines, evidenced by short branch lengths in the phylogeny. Some accessions shared the same cultivar name but were donated by different sources and maintained as separate clones in the CePaCT genebank. For example, multiple copies of ‘Wanmum Small’, ‘Igi’, and ‘Keania’ were donated to CePaCT by the USDA genebank in Griffin, GA, and the Plant Breeding Institute (PBI) in Burney, Australia. These accessions clustered together by variety name and appeared genetically similar. Additionally, CePaCT held three accessions named ‘Tongamai’ and ‘Halasiki’, donated by three different institutions, but these appeared to be genetically identical. The ‘Tongamai’ accession from CePaCT clustered with material from CIP in the Peru/Ecuador cluster and was genetically like CIP 440276, also named ‘Tongamai’.

**Figure 9:**
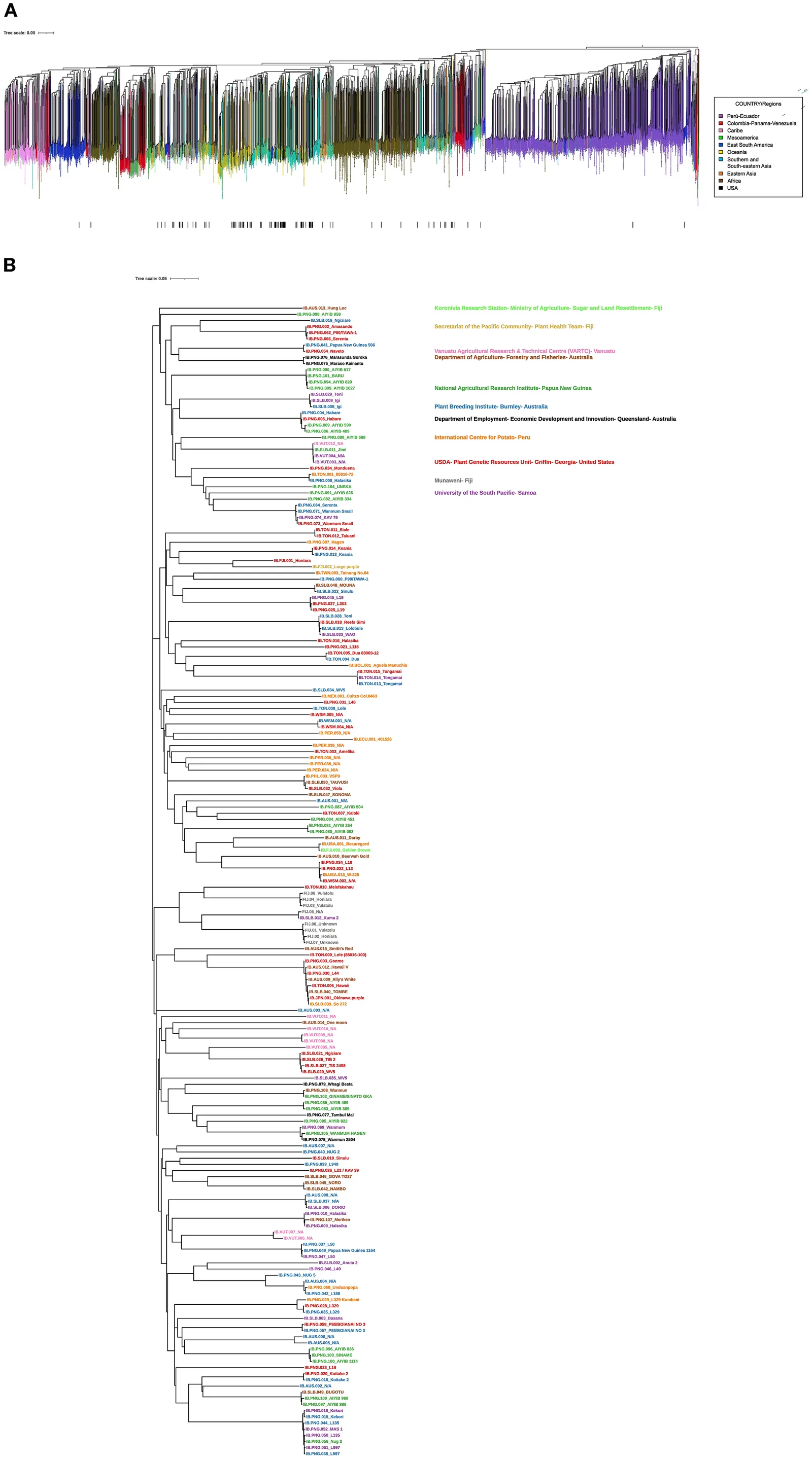
Phylogenetic trees of **(A)** CePaCT sweetpotato accessions and CIP accessions, black bars below the tree indicate the position of CePaCT accessions **(B)** CePaCT accessions only.

Looking at the history of the CePaCT collection, it was discovered that the University of the South Pacific (USP), Samoa, and PBI materials were collected during the European Union (EU)-funded Pacific Regional Agricultural Programme (PRAP) during the 1990s. These materials were then donated to CePaCT for conservation; however, the accessions were not pathogen-tested. To facilitate distribution, pathogen-tested analogues were obtained from the USDA. Both sets of the same accessions were retained. Genotyping has clearly identified redundancies within the CePaCT collection, and tracing the history of the material helps explain why these redundancies exist. The initial goal of the PRAP project was to collect, evaluate, and distribute the material to farmers within the Pacific. However, a lack of pathogen-testing capacity in the region meant the collections were not tested and remained conserved at CePaCT. CePaCT then obtained pathogen-tested material for distribution but did not remove the untested material from the collection, leading to multiple copies of the same genotype. This was primarily because there was no clear evidence that the pathogen-tested material was genetically similar to the material conserved at CePaCT. The current study clearly identifies these redundancies and provides CePaCT with an opportunity to rationalize aspects of the collection and improve management efficiency. It also highlights the need to collect further Pacific diversity, which remains unrepresented in both the CePaCT and broader CIP collections.

The 188 accessions from CePaCT were added to the tree of the entire CIP collection to assess differences between the two collections. A total of 30 unique accessions were identified from the CePaCT sweetpotato collection that were not part of the CIP collection. These accessions represent genetic gaps in the CIP collection and could be targeted for acquisition to fill these gaps. It is relatively straightforward for one genebank to share material with another, compared to obtaining permission to collect germplasm within a country to address genetic gaps. Sixty-one accessions from CePaCT were not identified as originating from CIP, but they genotypically matched material in the CIP collection, indicating some genetic overlap between the two collections. Overall, the majority of the CePaCT material clustered with accessions from Asia, Oceania, and Africa in the CIP collection, which was expected given that most of the material was donated or collected from these regions.

### 3.3 A sweetpotato core collection

The size and heterogeneity of most germplasm collections often hinder their use in plant improvement (Brown, 1989). Core collections, a 10% subgroup maximizing a representative or the diversity in the entire collection, are valuable tools for managing germplasm, particularly when it comes to efficiently utilizing large collections for plant breeding. These collections are designed to represent the majority of the genetic diversity within a germplasm collection, with minimal redundancy, all contained within a much smaller subset than the entire collection. Stratified sampling of grouped accessions is the most effective strategy for selecting a core collection based on available data, which can include geographical origin, genetic characteristics, and breeding value (Brown, 1989). Core collections allow researchers to assess a smaller, diverse subset, making it more economical to evaluate important traits compared to screening an entire collection. Core collections have been widely used to screen for traits of interest in breeding programs and to identify additional accessions within the larger germplasm collection that may contain those traits.

The DArTseq genotyping data were used to construct a cultivated sweetpotato core collection using Core Hunter version 3 (De Beukelaer et al., 2018), along with manual selections made by the sweetpotato curator. Highly similar accessions were removed from the dataset, as described previously. A total of 3, 431 non-redundant cultivated accessions were evaluated, including breeding lines, improved varieties, landraces, and five accessions with an unknown biological status. A stratified sampling strategy was selected considering the phylogeny and genetic structure to identify genetic groups or clusters. After identifying the genetic groups, the size of the sample selected for the core collection was proportional to the diversity of each group, and expected heterozygosity was calculated within each group or cluster. The *sampleCore* function was used to specify the number of accessions to be selected.

A total of 342 accessions were selected for the core collection, representing 9.97% of the non-redundant portion of the germplasm collection (3, 431 accessions). Typically, core collections target 10-15% of the total collection. **Figure 10A** illustrates the proportion of samples and their origins in the dataset, along with the selected core collection. A PCoA was performed to assess the representativeness of the core compared to the entire germplasm collection. **Figure 10B** shows the first three axis of variation among the non-redundant accessions and the 342 core accessions, demonstrating that genetic diversity was maximized within the core subset. A phylogenetic tree of the accessions was constructed (**Figure 10C**), showing that the core collection was well-represented across each cluster in the phylogeny. The clusters containing Peruvian and African material had fewer selections in the core, due to higher levels of genetic redundancy in these materials, primarily because of a high proportion of similar breeding lines.

**Figure 10:**
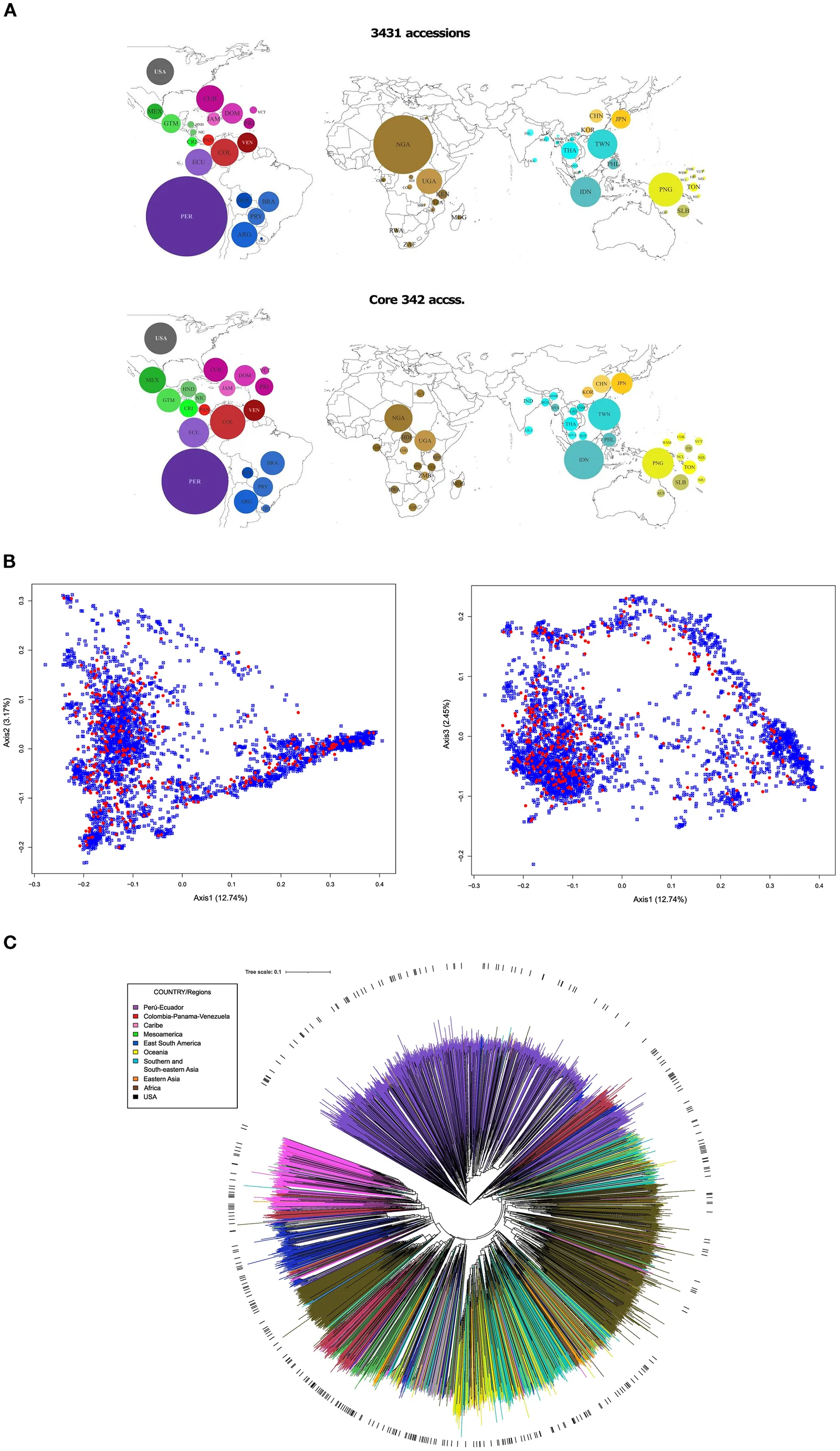
**(A)** Country distribution of the initial set of 3,431 unique accessions (top panel) and the generated core collection of 342 accessions (bottom panel). The size of the circle is proportional to the number of accessions within each group. **(B)** Principal Coordinate Analysis. Blue squares represent the total set of 3, 431 accessions, while the red points represent the selected core of 342 accessions. Left image axis 1 vs axis 2 and right image axis 1 vs axis 3. **(C)** The core collection composed of 342 accessions, is indicated by short bars in the outer ring of the 3, 431 accession Neighbor-Joining Tree, colored by geographic regions.

## 4 Conclusion

Fingerprinting entire germplasm collections using NGS approaches enhances the value of genetic resource collections for both the genebank and its user. This molecular data will serve as a valuable resource for the sweetpotato community for years to come. It can be combined with traditional passport and phenotyping data to improve sweetpotato breeding efforts and enable more targeted and strategic selections of germplasm for research. While relatively few studies have focused on collection-wide fingerprinting, especially in large germplasm collections, the decreasing cost of NGS data generation will likely make this approach more common in the future. Several projects are already conducting collection-wide genotyping. Such comprehensive data can offer valuable insight, including an understanding of inter- and intraspecific relationships among accessions within a collection. Understanding genetic diversity, population structure, and genetic redundancies within large collections, like the CIP sweetpotato collection, greatly aids in managing the collection. It allows for the identification of genetic redundancy, which can help in archiving (CGIAR, 2022) or deprioritizing efforts on genetically redundant material to make room for more diverse material.

The genetic similarity thresholds adopted in this study should not be interpreted as defining biological clone identity or true genetic duplicates. High genetic similarity may result from different biological scenarios, including identical clones, closely related breeding materials, somaclonal variation, or other sources of genetic similarity. Consequently, the molecular analyses presented here should be regarded as a decision-support tool for germplasm rationalization, archiving, acquisition, and the prioritization of curatorial activities, rather than as the sole basis for deciding whether clonal accessions should be retained, eliminated, or archived. Integration of complementary passport, pedigree, morphological, historical, and curatorial information remains essential for confirming whether highly similar accessions should ultimately be considered redundant for long-term genebank management.

In the current study, approximately 42% of the collection (2, 499 accessions) were genetically similar and may need to be considered for archival. It is important to highlight, however, that archiving clonal accessions compared to seed is relatively complex, since the *in vitro* samples, in the case of sweetpotato, will die in about one year without being sub-cultured. This is particularly important in clonal collections, where the maintenance of each individual accession has significant cost and time required by staff to keep clones constantly propagated, true-to-type, and readily available, in contrast to seed collections. Fingerprinting newly acquired material can help make informed decisions before investing time and money into the introduction and cleaning of clonal material - an expensive and time-consuming process that often takes two to four years to be accomplished. Based on that CIP has introduced a practice of genotyping prior to formally adding new accessions to the collection to avoid the introduction of redundant materials. Population structure data also reveals information about hybridization and introgressions among various ancestral populations within the collection, providing insights that are not easily obtained from morphological assessments or other experiments. Furthermore, if genotyping data for all sweetpotato *ex situ* collections were widely available, comparisons could be made easily to identify unique and redundant material, which is often difficult to infer based on passport data, accession numbers, or cultivar names alone. In this study, sweetpotato collections were compared by genotyping a portion of the germplasm maintained at CePaCT. Overall, genotyping the entire collection allows for more efficient management, and provides the necessary information to make strategic decisions about what should be retained, eliminated, or acquired to create a well-represented and diverse collection. It also facilitated the establishment of a core collection with 342 sweetpotato accessions, which benefits the research community. Additionally, this genotyping data is now available for the breeding and research communities to support genome-wide association studies (GWAS), genomic selection (GS) studies, association mapping, and other applications.

## Data Availability

The datasets presented in this study can be found in online repositories in https://data.cipotato.org/dataset.xhtml?persistentId=doi:10.21223/P3/S2IMOS.
